# p21-Activated Kinases 1, 2 and 4 in Endometrial Cancers: Effects on Clinical Outcomes and Cell Proliferation

**DOI:** 10.1371/journal.pone.0133467

**Published:** 2015-07-28

**Authors:** Michelle K. Y. Siu, Daniel S. H. Kong, Sheila Y. P. Ngai, Hoi Yan Chan, Lili Jiang, Esther S. Y. Wong, Stephanie S. Liu, Karen K. L. Chan, Hextan Y. S. Ngan, Annie N. Y. Cheung

**Affiliations:** 1 Department of Pathology, The University of Hong Kong, Hong Kong, HKSAR, China; 2 Department of Obstetrics and Gynaecology, The University of Hong Kong, Hong Kong, HKSAR, China; Children's Hospital Boston, UNITED STATES

## Abstract

p21-activated kinases (Paks) are serine/threonine protein kinases involved in biological events linked to malignant tumor progression. In this study, expression of Pak1, p-Pak2 Ser^20^, Pak4, pPak4 Ser^474^ in 21 normal endometrium, 16 hyperplastic endometrium without atypia, 17 atypical complex hyperplasia and 67 endometrial cancers was assessed by immunohistochemistry and correlated with clinicopathological parameters. We also accessed the proliferative role and downstream targets of Pak1 in endometrial cancer. Pak1 was expressed in cytoplasm whereas Pak4 and p-Pak4 were expressed in both cytoplasm and nucleus of endometrial tissues. In normal endometrium, significantly higher Pak1 (*P* = 0.028) and cytoplasmic p-Pak2 (*P* = 0.048) expression was detected in proliferative endometrium than secretory endometrium. Pak1, cytoplasmic and nuclear Pak4 and nuclear p-Pak4 was significantly overexpressed in endometrial cancer when compared to atrophic endometrium (all *P*<0.05). Moreover, type I endometrioid carcinomas showed significantly higher Pak1 expression than type II non-endometrioid carcinomas (*P*<0.001). On the other hand, Pak1, Pak4 and p-Pak4 expression negatively correlated with histological grade (all *P*<0.05) while p-Pak2 and cytoplasmic Pak4 expression inversely correlated with myometrial invasion (all *P*<0.05). Furthermore, patients with endometrial cancers with lower cytoplasmic Pak4 expression showed poorer survival (*P* = 0.026). Multivariate analysis showed cytoplasmic Pak4 is an independent prognostic factor. Functionally, knockdown of Pak1, but not Pak4, in endometrial cancer cell line led to reduced cell proliferation along with reduced cyclin D1, estrogen receptor (ERα) and progestogen receptor (PR) expression. Significant correlation between Pak1 and PR expression was also detected in clinical samples. Our findings suggest that Pak1 and cytoplasmic p-Pak2 may promote cell proliferation in normal endometrium during menstral cycle. Pak1, cytoplasmic and nuclear Pak4 and nuclear p-Pak4 are involved in the pathogenesis of endometrial cancer especially in postmenopausal women. Pak1 promote endometrial cancer cell proliferation, particular in type I endometrioid carcinoma. Cytoplasmic Pak4 can be potential prognostic marker in endometrial cancer.

## Introduction

Endometrial cancer is the most common gynecological malignancy worldwide [1] and its incidence in Asia is rising. It can be classified into two major clinicopathological types. Estrogen dependent type I endometrioid carcinoma, comprises about 80% of endometrial cancers, is usually associated with endometrial hyperplasia [2, 3]. Type II non-endometroid carcinoma, which is estrogen-independent and includes serous carcinoma and clear cell carcinoma, usually occurs in atrophic endometrium [2, 3]. Type I endometrioid carcinoma has favorable prognosis while type II endometrial carcinoma is more aggressive [2, 3].

p21-activated serine/threonine kinases (Paks) are effectors for the small Rho GTPases Rac1 and Cdc42 that play important roles in a variety of cellular functions, including cell morphogenesis, motility, survival, anchorage-independent growth and angiogenesis, all of which are prerequisite steps for tumor formation and tumor invasion [4, 5]. Based on different domain structures and biochemical properties, the six members of Pak family are classified into Group I (Paks1-3) and Group II (Paks 4–6), which have different expression pattern, substrate priority and functional specificity in various tissues [5–7].

We have recently identified Pak1 and Pak4 as prognostic markers and potential therapeutic targets for ovarian cancer [8, 9]. Increased phosphorylated (p)-Pak2^Ser20^ expression in ovarian cancer was also detected [9]. Pak1 and Pak4 promote ovarian cancer cell migration and invasion through the p38/VEGF pathway and the c-Src/MEK-1/MMP2 pathway, respectively [8, 9]. Pak4 also induce ovarian cancer cell proliferation through the Pak4/c-Src/EGFR/cyclin D1/CDC25A pathway [8]. We also revealed overexpression of Pak1 in choriocarcinoma and hydatidiform moles that progressed to aggressive disease [10]. Pak1 regulate choriocarcinoma cell proliferation, migration and invasion ability in association with altered level of p16, VEGF and MT1-MMP [10].

Up-regulation of Pak1 and Pak4 has also been observed in other human cancers [4, 5, 11], such as breast and colon cancers. A recent study has reported that Pak1 is down-regulated by progesterone during the secretory phase in normal endometrium [12]. However, differential expression of Paks in endometrial carcinoma is limited. Herein, the expression and localization of Pak1, phosphorylated (p)-Pak2^Ser20^, Pak4 and p-PAK4^Ser474^ (the activated form) in normal, hyperplastic and cancerous endometrium was assessed and correlated with clinicopathological parameters. The proliferative role and downstream targets of Pak1 in endometrial cancer was further investigated.

## Materials and Methods

### Clinical samples

Sixty-seven formalin fixed, paraffin embedded samples of endometrial cancers including 56 endometrioid carcinomas, 7 serous carcinomas and 4 clear cell carcinomas were retrieved from Department of Pathology, the University of Hong Kong, Queen Mary Hospital for immunohistochemistry. Clinical and pathological parameters including estrogen receptor alpha (ERα) and progesterone receptor (PR) status [13] as assessed by immunohistochemistry was retrieved. Twenty-one samples of normal endometrium (including 10 atrophic, 5 secretory and 6 proliferative endometrium), 16 samples of hyperplastic endometrium without atypia (including 7 simple hyperplasia and 9 complex hyperplasia) and 17 samples of atypical complex hyperplasia were also retrieved. Among the 17 atypical complex hyperplasia, 8 cases were pure atypical hyperplasia while 9 cases subsequently developed endometrial cancers (atypical hyperplasia with endometrial cancers). The average age of these patients was 57.2 years (range 20–82 years). The mean follow-up period was 42.75 months (range 1–108 months). Written informed consent was obtained by all patients. This study was approved by the Institutional Ethical Review Board of the University of Hong Kong/Hospital Authority Hong Kong West Cluster (HKU/HA HKW IRB). Haematoxylin Eosin sections were examined by pathologists to review the diagnosis and to ensure that each sample is composed of more than 75% tumor cells.

### Cell lines

A human normal endometrial cell line (NEM), extended from primary culture of normal endometrial cells from a ~ 40-year old premenopausal female with regular 28-day menstrual cycles, was kindly given by Prof. Doris Benbrook (University of Oklahoma Health Sciences Center) [14]. Three human endometrial cancer cell lines, HEC-1B, HEC-1A and RL95-2 were from ATCC (Manassas, VA). All culture were cultured as previously described [15].

### Transient knockdown of Pak1 and Pak4 in RL95-2

Cells were transfected with 100 nM each of si*GENOME* Smart-pool for Pak1, Pak4 and si*Control* nontargeting siRNA pool (Dharmacon, Lafayette, CO), using SilentFect (Bio-Rad Laboratories, Hercules, CA) per manufacturer’s instructions for 48 hours before RNA and protein extraction, cell counting and cell plating [10].

### Immunohistochemistry

Briefly, formalin-fixed paraffin-embedded sections were stained with antibodies against Pak1, p-Pak2^Ser20^, Pak4 and p-Pak4 Ser^474^ (as listed in S1 Table) using EnVision+ Dual Link System (K4061; Dako, Carpinteria, CA) [8–10]. Microwave antigen recovery was performed using citrate buffer (pH 6.0). Omission or substitution of the primary antibody with preimmune IgG serum was used as a negative control. Both the intensity and percentage of stained epithelial cells were evaluated. Immunoreactivity was evaluated semiquantitatively by intensity and percentage of epithelium stained. Staining intensity was scored as 0 (negative), 1 (weak), 2 (mild), 3 (moderate) and 4 (strong). The percentage of positive cells was rated as 0 (<5%), 1 (5%-25%), 2 (26%-50%), 3 (51%-75%) and 4 (>75%). A composite “Histoscore” was given by multiplying the staining intensity (0–4) by the percentage of stained cells (0–4) with a maximum score of 16 [16].

### Real-time PCR

Total RNA was extracted from cancer cell lines using Trizol reagent (Invitrogen). 2.5 μg total RNA was reverse transcribed by SuperScript Reverse Transcriptase (Invitrogen, San Diego, CA). Real-time PCR was performed with ABI Prism 7700 sequence detection system (Applied Biosystems, Foster City, CA) [17, 18]. Primer sequences for Pak1, Pak4, CDC25A, cyclin D1, ERα, PR (target both PRA and PRB isoforms) and GAPDH (as internal control) were shown in S2 Table. The PCR purity was confirmed by gel electrophoresis.

### Immunoblotting

Cells were harvested with lysis buffer (0.125 M Tris, pH 6.8 at 22 oC containing 1% NP-40 (v/v), 2 mM EDTA, 2 mM N-ethylmaleimide, 2 mM PMSF, 1 mM sodium orthovanadate and 0.1 μM sodium okadate], and cleared by centrifugation at 4°C. Protein concentration was determined by DC (detergent compatible) protein assay (Bio-Rad Laboratories, Hercules, CA). 20 μg protein was resolved by SDS-PAGE, transferred to polyvinylidene difluoride membrane, and hybridized with corresponding antibodies (as listed in S1 Table) [17, 18].

### Cell proliferation was determined by cell count method

3 x 10^4^ cells were seeded in 6-well culture plates. Cell number was counted using trypan blue dye exclusion with hematocytometer at day 4 [18].

### Statistical Analysis

Statistical analysis was performed using SPSS 15.0 for Windows (SPSS Inc., Chicago, IL). Mann-Whitney test was applied for comparison between two groups. Kruskal-Wallis rank test was applied for comparison among multiple groups. Survival analysis was performed by Kaplan-Meier analysis and log-rank test. *P* values < 0.05 were considered as statistically significant.

## Result

### Expression of Pak1 and cytoplasmic p-Pak2 Ser^20^ were higher in proliferative endometrium. Pak1, cytoplasmic and nuclear Pak4 and nuclear p-Pak4 Ser^474^ were overexpressed in endometrial cancer when compared with atrophic endometrium

**Fig 1:**
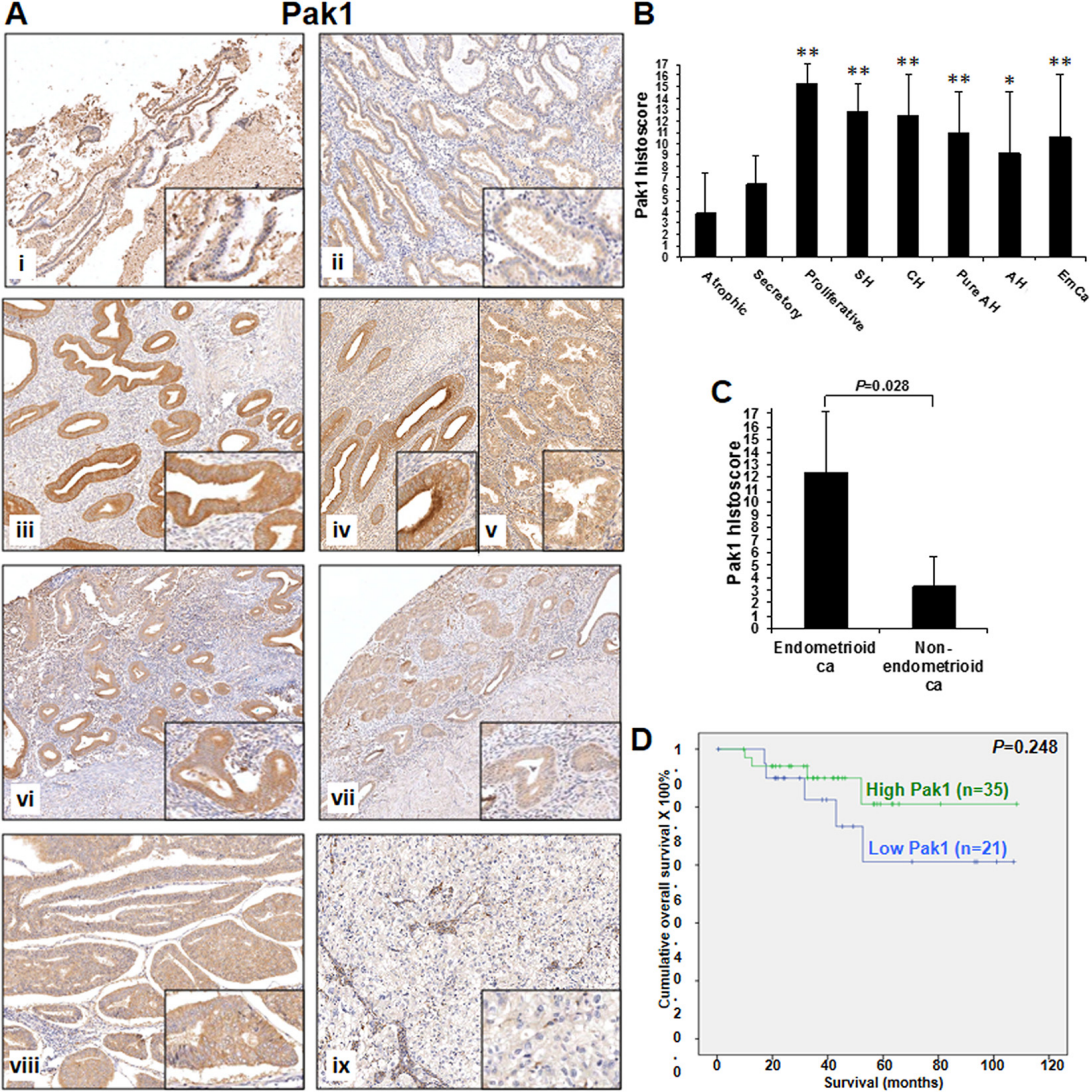
(A) Pak1 immunoreactivity in normal atrophic endometrium (i), secretory endometrium (ii), proliferative endometrium (iii), simple hyperplasia (iv), complex hyperplasia (v), pure atypical hyperplasia (vi), atypical hyperplasia (vii), endometrioid carcinomas (viii) and non-endometrioid carcinomas (ix). (B) Bar chart showing the immunoreactivity of Pak1 in normal atrophic endometrium (Atrophic), secretory endometrium (Secretory), proliferative endometrium (Proliferative), simple hyperplasia (SH), complex hyperplasia (CH), pure atypical hyperplasia (Pure AH), atypical hyperplasia (AH) and endometrial cancers (EmCa). *, *P*<0.05; **, *P*<0.005; compared to normal atrophic endometrium by Mann-Whitney test. (C) Bar chart showing the immunoreactivity of Pak1 in endometrioid carcinomas (ca) and non-endometrioid carcinomas. The immunoreactivity of Pak1 was analyzed by Mann-Whitney test. (D) Kaplan-Meier survival curves for endometrial cancer patients with two different expression levels of Pak1 (cut off at mean).

**Fig 2:**
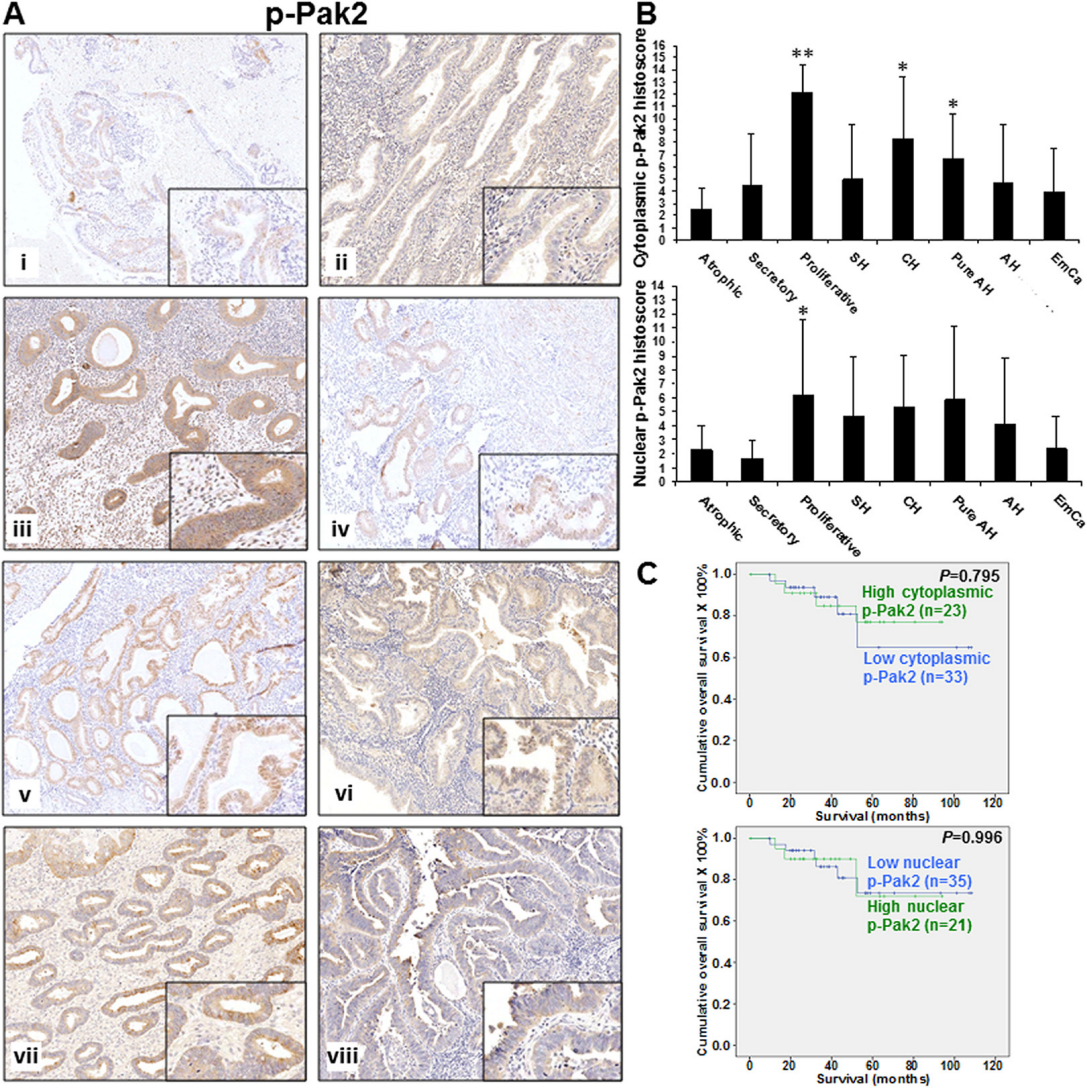
(A) p-Pak2 immunoreactivity in normal atrophic endometrium (i), secretory endometrium (ii), proliferative endometrium (iii), simple hyperplasia (iv), complex hyperplasia (v), pure atypical hyperplasia (vi), atypical hyperplasia (vii) and endometrial cancers (viii). (B) Bar chart showing the immunoreactivity of cytoplasmic (upper panel) and nuclear (lower panel) p-Pak2 in normal atrophic endometrium (Atrophic), secretory endometrium (Secretory), proliferative endometrium (Proliferative), simple hyperplasia (SH), complex hyperplasia (CH), pure atypical hyperplasia (Pure AH), atypical hyperplasia (AH) and endometrial cancers (EmCa). *, *P*<0.05; **, *P*<0.005; compared to normal atrophic endometrium by Mann-Whitney test. (C) Kaplan-Meier survival curves for endometrial cancer patients with two different expression levels of cytoplasmic (upper panel) and nuclear (lower panel) p-Pak2 (cut off at mean).

By immunohistochemistry, Pak1 was predominately expressed in the cytoplasm of endometrial tissues with only focal nuclear staining detected (Fig 1A). In normal endometrial samples, atrophic and secretory endometrium showed weak expression whereas proliferative endometrium displayed strong expression (Fig 1A i-iii). Hyperplastic endometrium and endometrial cancers showed moderate to strong expression (Fig 1A iv-ix). Pak1 expression in proliferative endometrium was significantly higher than that in atrophic (*P* = 0.001) and secretory (*P* = 0.004) endometrium, atypical hyperplasia (*P* = 0.042) and endometrial cancers (*P* = 0.044) (Fig 1B). Significantly higher Pak1 expression in hyperplastic endometrium and endometrial cancers than in atrophic endometrium (both *P*<0.05) was observed (Fig 1B). Although higher Pak1 expression was found in hyperplastic endometrium and endometrial cancers than secretory endometrium, significant difference was only reached between simple/complex hyperplasia and secretory endometrium (Fig 1B). Similar Pak1 expression was detected in hyperplastic endometrium and endometrial cancers.

p-Pak2 was detected in both cytoplasm and nucleus of endometrial tissues (Fig 2A). Among all categories, proliferative endometrium showed the strongest cytoplasmic and nuclear p-Pak2 expression (Fig 2A). Atrophic and secretory endometrium as well as endometrial cancers showed weak cytoplasmic and nuclear p-Pak2 expression whereas hyperplastic endometrium showed moderate cytoplasmic and nuclear p-Pak2 expression (Fig 2A). Cytoplasmic p-Pak2 expression in proliferative endometrium was significantly higher than that in all other categories (all *P*<0.05) except complex hyperplasia whereas nuclear p-Pak2 only showed significantly higher expression in proliferative endometrium than atrophic (*P* = 0.046) and secretory (*P* = 0.028) endometrium and endometrial cancers (*P* = 0.007) (Fig 2B). Although higher cytoplasmic and nuclear p-Pak2 expression was found in hyperplastic endometrium than atrophic endometrium, only complex hyperplasia (*P* = 0.007) and pure atypical hyperplasia (*P* = 0.013) showed significant higher cytoplasmic p-Pak2 expression (Fig 2B). Furthermore, there was significant reduced cytoplasmic and nuclear p-Pak2 expression in endometrial cancers than that in complex hyperplasia (*P* = 0.009 and *P* = 0.006, respectively) and pure atypical hyperplasia (*P* = 0.038 and *P* = 0.042, respectively) (Fig 2B).

**Fig 3:**
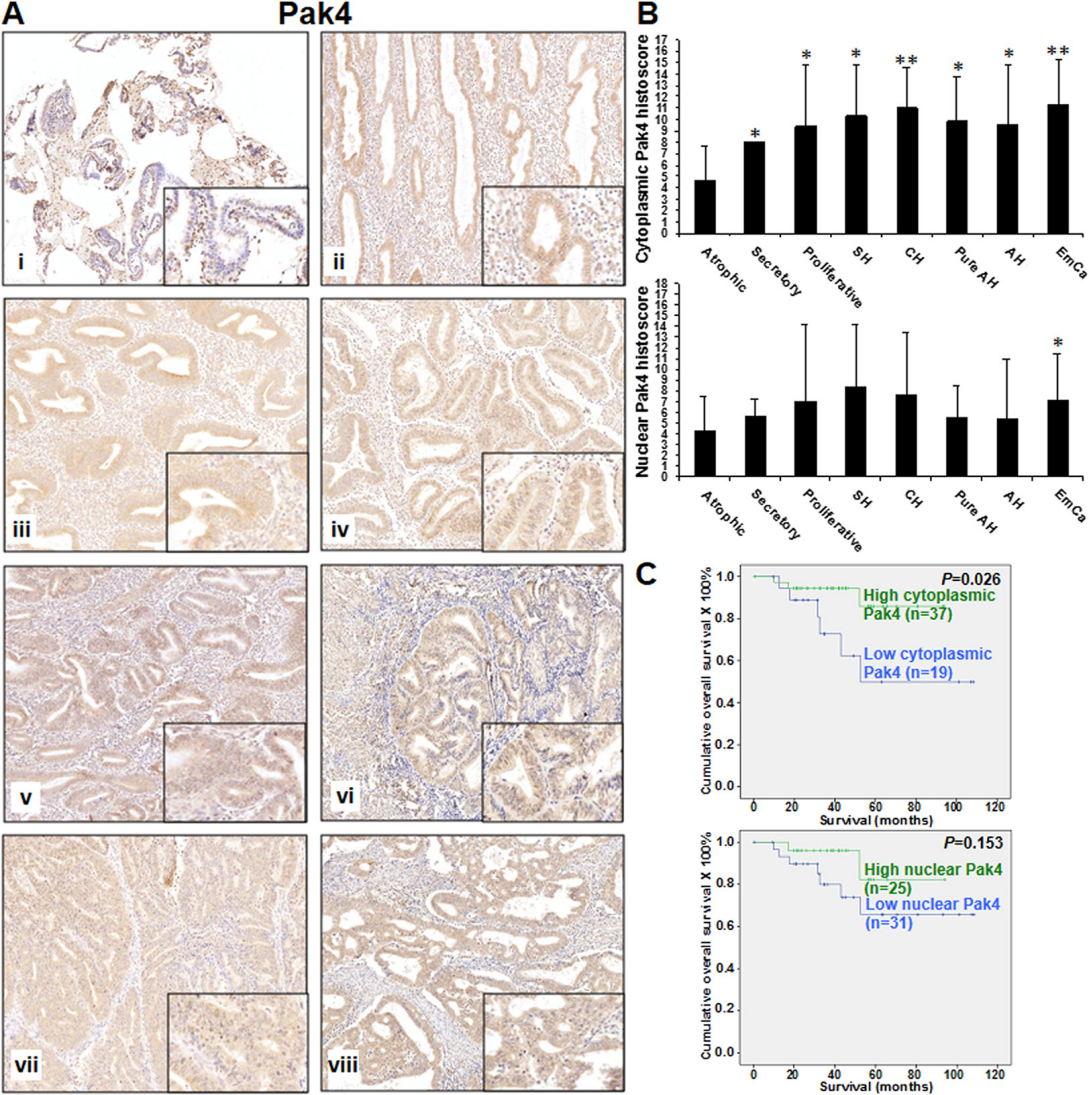
(A) Pak4 immunoreactivity in normal atrophic endometrium (i), secretory endometrium (ii), proliferative endometrium (iii), simple hyperplasia (iv), complex hyperplasia (v), pure atypical hyperplasia (vi), atypical hyperplasia (vii) and endometrial cancers (viii). (B) Bar chart showing the immunoreactivity of cytoplasmic (upper panel) and nuclear (lower panel) Pak4 in normal atrophic endometrium (Atrophic), secretory endometrium (Secretory), proliferative endometrium (Proliferative), simple hyperplasia (SH), complex hyperplasia (CH), pure atypical hyperplasia (Pure AH), atypical hyperplasia (AH) and endometrial cancers (EmCa). *, *P*<0.05; **, *P*<0.005; compared to normal atrophic endometrium by Mann-Whitney test. (C) Kaplan-Meier survival curves for endometrial cancer patients with two different expression levels of cytoplasmic (upper panel) and nuclear (lower panel) Pak4 (cut off at mean).

**Fig 4:**
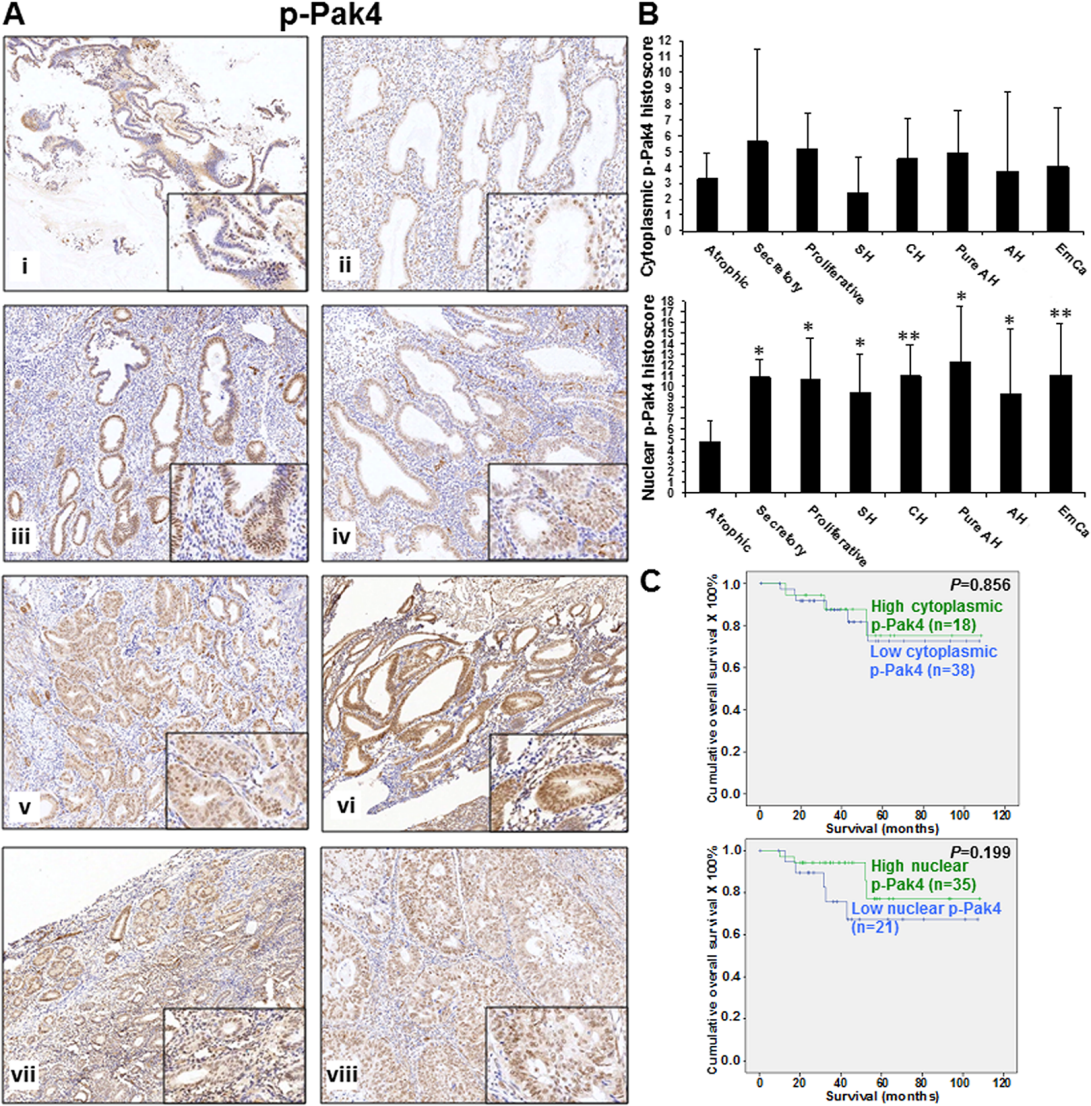
(A) p-Pak4 immunoreactivity in normal atrophic endometrium (i), secretory endometrium (ii), proliferative endometrium (iii), simple hyperplasia (iv), complex hyperplasia (v), pure atypical hyperplasia (vi), atypical hyperplasia (vii) and endometrial cancers (viii). (B) Bar chart showing the immunoreactivity of cytoplasmic (upper panel) and nuclear (lower panel) p-Pak4 in normal atrophic endometrium (Atrophic), secretory endometrium (Secretory), proliferative endometrium (Proliferative), simple hyperplasia (SH), complex hyperplasia (CH), pure atypical hyperplasia (Pure AH), atypical hyperplasia (AH) and endometrial cancers (EmCa). *, *P*<0.05; **, *P*<0.005; compared to normal atrophic endometrium by Mann-Whitney test. (C) Kaplan-Meier survival curves for endometrial cancer patients with two different expression levels of cytoplasmic (upper panel) and nuclear (lower panel) Pak4 (cut off at mean).

Pak4 was expressed in both cytoplasm and nucleus of endometrial tissues (Fig 3). Cytoplasmic and nuclear Pak4 showed weak expression in atrophic endometrium and moderate to strong expression in secretory, proliferative, hyperplastic endometrium and endometrial cancers (Fig 3). Cytoplasmic Pak4 expression in atrophic endometrium was significantly lower than that in other categories (all *P*<0.05) (Fig 3B, upper panel). Significantly higher cytoplasmic Pak4 expression was also found in secretory endometrium than complex hyperplasia (*P* = 0.047) and endometrial cancers (*P* = 0.036) (Fig 3B, upper panel). Nuclear Pak4 in atrophic endometrium also displayed lower expression than other categories. However, significant difference of nuclear Pak4 expression only observed between endometrial cancers and atrophic endometrium (*P* = 0.026) (Fig 3B, lower panel). There is no significant difference of cytoplasmic and nuclear Pak4 expression between other categories.

p-Pak4 was also detected in both cytoplasm and nucleus of endometrial tissues (Fig 4A). Cytoplasmic p-Pak4 showed weak to moderate expression in all categories (Fig 4A) and significant difference was only found between proliferative endometrium and simple hyperplasia (*P* = 0.041) (Fig 4B, upper panel). Weak to moderate nuclear p-Pak4 expression was observed in atrophic endometrium whereas other categories displayed strong expression (Fig 4A). Significant difference was found between atrophic endometrium and other categories (all *P*<0.05) (Fig 4B, lower panel). Since the present study involved 6 proliferative endometrium cases only, the error bar for nuclear pPak2 and Pak4 in proliferative endometrium is relatively large.

**Fig 5:**
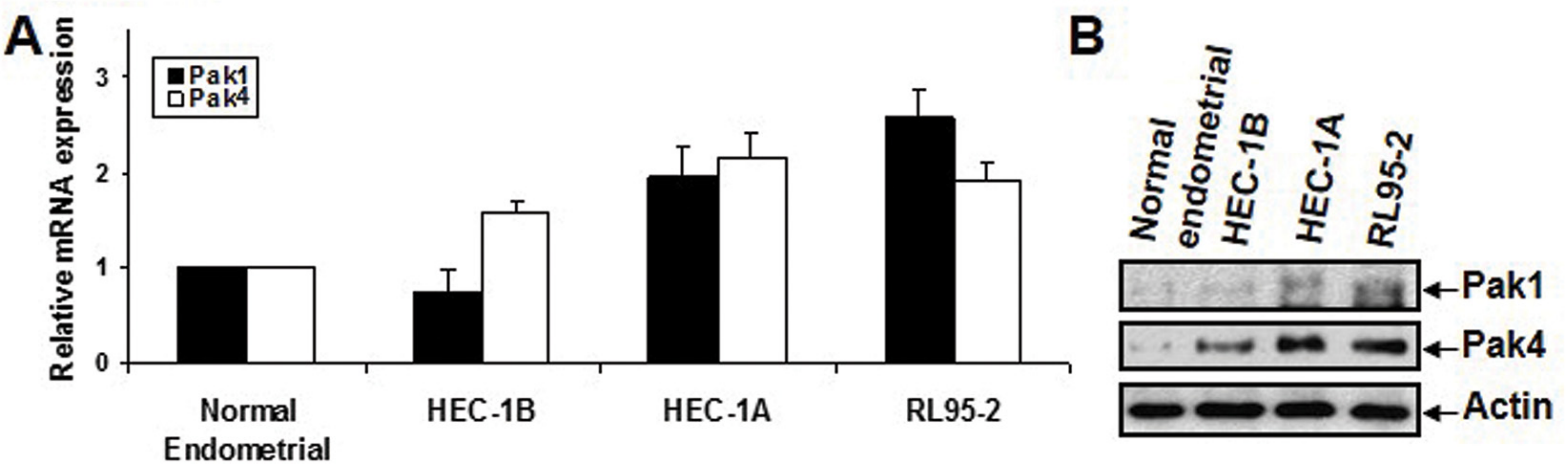
mRNA (A) and protein (B) expression of Pak1 and Pak4 in a human normal endometrial cell line, and three endometrial cancer cell lines, HEC-1B, HEC-1A and RL95-2, as determined by qPCR and immunoblot analysis respectively.

Up-regulation of Pak1 and Pak4 mRNA and protein expression was also found in cancer cell lines (HEC-1B, HEC-1A and RL95-2) compared with normal endometrial cells by qPCR (Fig 5A) and Western blot analysis (Fig 5B), respectively.

### Pak1, Pak4 and p-Pak4 expression correlated with clinicopathological parameters. Pak1 expression correlated with PR

Pak1 expression was found to correlate significantly with histological type, histological grade (grade 1/2 vs. 3) and patients’ age at diagnosis (all *P*<0.05), but not with other clinical parameters (S3 Table). Patients with type I endometrioid carcinoma (Fig 1C), Grade 1 and 2 and age ≤ 57 displayed higher Pak1 expression than those with type II serous and clear cell carcinoma, grade 3 and age > 57. Significantly higher cytoplasmic and nuclear p-Pak2 as well as cytoplasmic Pak4 expression was observed in superficial myometrial invasion than deep myometrial invasion (all *P*<0.05) (S3 Table). There was a significant correlation existed between Pak4 and p-Pak4 expression (both cytoplasmic and nuclear) and histological grade (all *P*<0.05) (S3 Table). Higher grade of tumors showed lower expression of Pak4 and p-Pak4. Cytoplasmic Pak4 showed significantly lower expression in patients with late stage disease and cancers involving cervix (all *P*<0.05) (S3 Table). No significant correlation between expression of p-Pak2, Pak4 and p-Pak4 with other clinical parameters was found.

Kaplan-Meier-survival analyses revealed that lower expression of cytoplasmic Pak4 (*P* = 0.026), but not Pak1, nuclear and cytoplasmic p-Pak2, nuclear Pak4, nuclear and cytoplasmic p-Pak4, resulted in a poorer survival in endometrial cancer patients (Figs 1D, 2C, 3C and 4C). Multivariate analysis revealed that cytoplasmic Pak4 is an independent prognostic factor (*P* = 0.037).

### Knockdown of Pak1 in endometrial cancer cells reduced cell proliferation and down-regulated cyclin D1, ERα and PR

**Fig 6:**
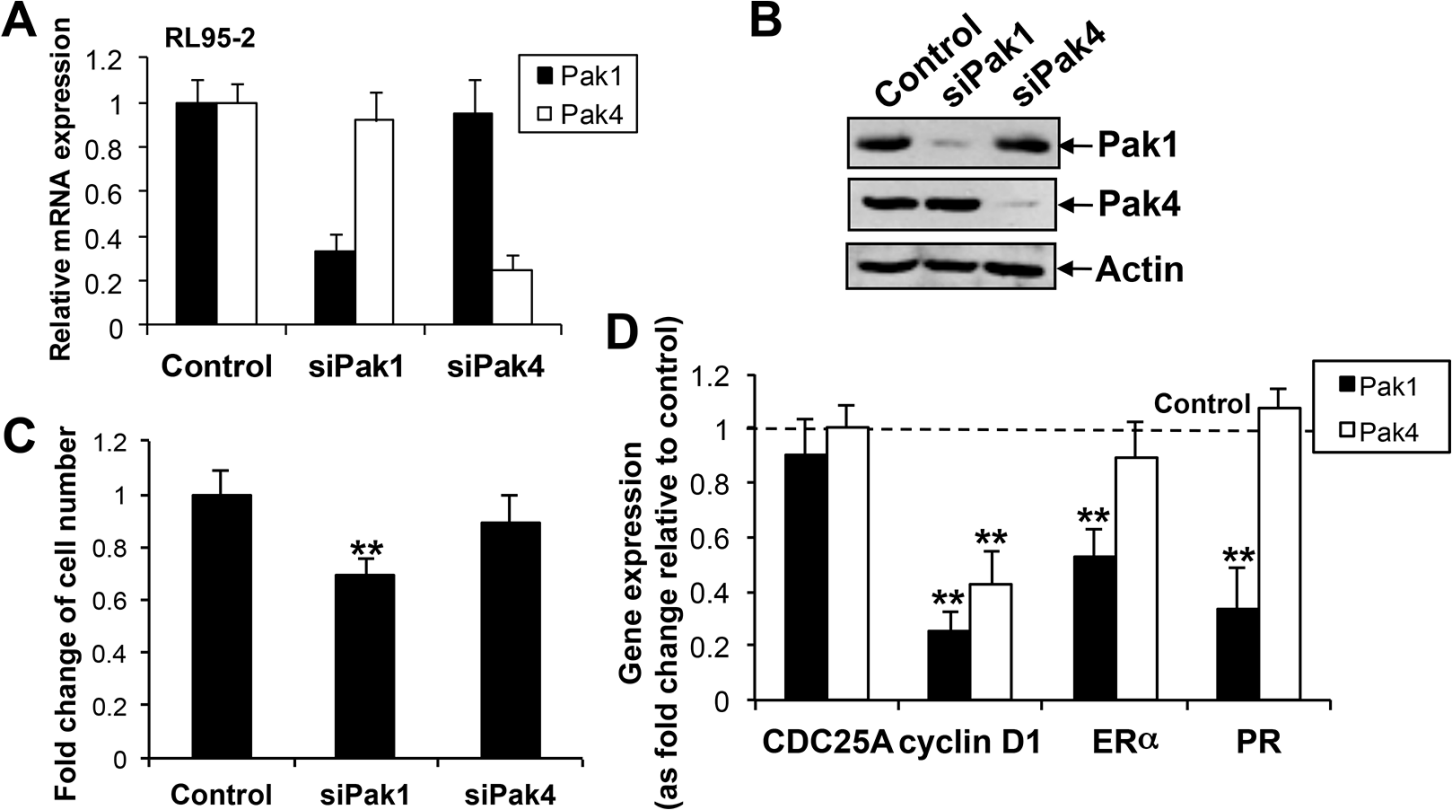
Transient knockdown of Pak1 (siPak1) and Pak4 (siPak4) mRNA and protein expression by qPCR (A) and Western blot analysis (B) respectively in RL95-2. (C) Cell proliferation rate of RL95-2 in control, siPak1 and siPak4 after 4 days was displayed as fold change compared to control; n = 3; **, *P*<0.005. (D) qPCR analysis on the mRNA expression of CDC25A, cyclin D1, ERα and PR in control, siPak1 and siPak4 RL95-2. Bars, means±SD of three experiments; **, *P*<0.005, Mann-Whitney test.

To determine the proliferative role of Pak1 and Pak4 in endometrial cancer cells, transient knockdown of Pak1 and Pak4 in RL95-2 was performed by siRNA approach. After confirming the specific knockdown of Pak1 and Pak4 mRNA (Fig 6A) and protein (Fig 6B), the effect on cell proliferation was determined by cell count method. Pak1 depleted RL95-2 showed significantly reduced proliferation rate when compared with control cells that transfected with control siRNA (Fig 6C). However, no significant difference in the proliferation rate after knockdown of Pak4 in RL95-2 was found (Fig 6C). Moreover, knockdown of Pak1 in RL95-2 significantly down-regulated cell cycle-related gene cyclin D1 and steroid receptors, ER**α** and PR, but have no effect on another cell cycle-related gene CDC25A, as evaluated by qPCR (Fig 6D). Cyclin D1 was also significantly reduced in siPak4 RL95-2 (Fig 6D).

## Discussion

Our data showed significantly higher Pak1 and cytoplasmic p-Pak2 expression in normal proliferative endometrium than secretory endometrium, suggesting Pak1 and p-Pak2 may be associated with estrogen regulated proliferative activity of endometrium during menstral cycle. In breast cancer, regulation of Pak1 by estrogen has been reported [19]. The findings also concur with previous report down-regulation of Pak1 by progesterone in normal secretory endometrium [12].

The proliferative role of Pak1 and Pak2 has been documented in normal cells. For example, in fibroblasts, Pak1 phosphorylated by CDC2A has been found to alter post-mitotic spreading, which is an essential step for cell cycle progression and proliferation [20]. Our previous study also revealed the involvement of Pak1 in trophoblast proliferation [10]. In normal mammary epithelial cells, hyperactive Rac3 was found to enhance cell proliferation through Pak2 [21]. Phosphorylation of Pak2 at Ser^20^, its corresponding phosphorylation site of Pak1 at Ser^20^ has been found to modulate Nck binding between cytosol and membrane, thus regulate cell migration [22, 23]. In our previous study, cytoplasmic p-Pak2 was detected in cytotrophoblast which can be considered as trophoblastic stem cells responsible for proliferation [10]. We also detected nuclear p-Pak2 in endometrial tissues, albeit the roles of p-Pak2 in nucleus still need to be further studied. Like Pak1 and Pak4 [8, 24], p-Pak2 in nucleus may play function on gene transcription. In future study, it is interest to determine if estrogen can regulate Pak1 and p-Pak2 expression leading to increased proliferative activity of endometrium during menstral cycle.

We found significantly higher Pak1, cytoplasmic and nuclear Pak4 and nuclear p-Pak4 expression in endometrial cancers than atrophic endometrium. Increased expression of Pak1 and Pak4 was also detected in endometrial cancer cell lines. Most functions of Pak4 are dependent on its kinase activity [8]. In our previous study, besides cell migration executed in the cytoplasm, we have revealed regulation of gene transcription by nuclear Pak4 in ovarian cancer [8]. The present findings suggest that Pak1, cytoplasmic and nuclear Pak4 and nuclear p-Pak4 may play important roles in the pathogenesis of endometrial cancer especially in postmenopausal women and can be useful in the detection of endometrial carcinoma in small endometrial biopsy from postmenopausal women.

We also demonstrated decreased proliferation in endometrial cancer cells after Pak1 knockdown along with reduced cyclin D1 expression. We and others have documented proliferative role of Pak1 in other human cancers, such as breast cancer [25], squamous nonsmall cell lung carcinoma [26] and choriocarcinoma [10]. Overexpression of cyclin D1, a D-type cyclin regulating G1-S phase cell cycle progression, has been reported in endometrial cancer [27, 28]. Previous study also found that Pak1 regulates prolactin mediated cyclin D1 promoter activity in breast cancer [29]. The present findings suggest that Pak1 may regulate cell proliferation through the alteration of cell cycle progression. Since cyclin D1 can be an alternative target of therapy [30] and small-molecule inhibitors for the Pak family are being developed [7, 31], combined-targeted therapy may be achieved for targeting endometrial cancer. Although we have revealed proliferation enhancing effect of Pak4 on ovarian cancer [8] and detected reduced cyclin D1 expression in RL95-2 cells after Pak4 knockdown in the present study, no significant effect of Pak4 on endometrial cancer cell proliferation could be demonstrated when Pak4 was transiently silenced in RL95-2 cells in the present study. It is possible that effect on cell proliferation needs to be determined using stable knockdown cells with longer incubation time before doing cell count method. Such possibility should be considered in future study. The roles of Pak4 in endometrial cancer need to be further elucidated.

We demonstrated significantly higher Pak1 expression in Type I endometrioid carcinoma than Type II non-endometrioid carcinoma. Type I endometrioid carcinoma is an estrogen-dependent tumor [2, 3]. Although previous study has shown no effect on Pak1 expression after estrogen treatment in Ishikawa cells (a well-differentiated endometrial adenocarcinoma cell line) [12], we found ER**α** and ER target gene PR, was reduced after knockdown of Pak1 in RL95-2. Moreover, the positive correlation between Pak1 and PR expression in our endometrial cancer clinical samples further support their link. The strong association between ER**α** and PR expression and Type I endometrioid carcinoma as compared with Type II non-endometrioid carcinoma [32], explains the effectiveness of progestin therapy for Type I endometrioid carcinoma. In breast cancer cells, Pak1 inhibitor blocked ER transactivation functions and downregulated ER target product PR (both PRA and PRB isoforms) protein expression [33]. Moreover, Pak1 mediated phosphorylation of ER**α** at Ser305 has shown to enhance transactivation of ER**α** leading to up-regulation of ER-regulated genes, such as cyclin D1, which in turn promote hormone-independent growth of breast cancer cells [33–35]. It is possible that Pak1 is one of upstream mediators for ER**α** and PR expression in Type I endometrioid carcinoma and Pak1 may enhance growth of endometrial cancer cells through regulating ER**α**

We found lower expression of cytoplasmic Pak4 was associated with poorer prognosis in endometrial cancers. Earlier studies including ours have found higher Pak4 expression contributing to poor prognosis in patients of ovarian cancer [8] and oral squamous-cell carcinoma [36]. This relatively surprising association may be related to the unique biological environment of the endometrium, being a cyclical proliferative tissue sensitive to hormone regulation. The reason for such observation needs to be further studied in future.

In summary, Pak1 and cytoplasmic p-Pak2 may play roles in the proliferative activity of endometrium during menstral cycle. Pak1, cytoplasmic and nuclear Pak4 and nuclear p-Pak4 are involved in the pathogenesis of endometrial cancer and can be potential therapeutic targets especially in postmenopausal women. Pak1 enhance endometrial cancer cell proliferation particular in type I endometrioid carcinoma. Cytoplasmic Pak4 can be potential prognostic marker in endometrial cancer.

## Supporting Information

S1 Table Sources and working dilutions of antibodies used in this study. (DOC)

S2 Table Primers used for real-time PCR. (DOC)

S3 Table Correlation of Pak1, nuclear and cytoplasmic p-Pak2, Pak4 and p-Pak4 with clinicopathological parameters in endometrial cancer. (DOC)
