# Supplementary material for: p21-Activated Kinases 1, 2 and 4 in Endometrial Cancers: Effects on Clinical Outcomes and Cell Proliferation
Source: PLoS One. 2015 Jul 28;10(7):e0133467. doi: 10.1371/journal.pone.0133467 (PMC4517872; doi:10.1371/journal.pone.0133467)
Supplement: S1 Table — (DOC) [file pone.0133467.s001.doc]

**S1 Table.** Sources and working dilutions of antibodies used in this study.

| **Antibodies** | **Catalog #** | **Companies** | **Applications*** |
| --- | --- | --- | --- |
| Rabbit anti-Pak1 | 2602 | Cell Signaling Technology, Inc. (Beverly, MA) | IHC (1:40) |
| Rabbit anti-Pak1/2 | sc-881 | Santa Cruz Biotechnology Inc. (Santa Cruz, CA) | WB (1:400) |
| Rabbit anti-p-Pak2 Ser^20^ | 2607 | Cell Signaling Technology, Inc. | IHC (1:50) |
| Rabbit anti-Pak4 | 3242 | Cell Signaling Technology, Inc. | IHC (1:50),  WB (1:1000) |
| Rabbit anti-p-Pak4 Ser^474^ | 3241 | Cell Signaling Technology, Inc. | IHC (1:50),  WB (1:1000) |
| Goat anti-Actin | sc-1616 | Santa Cruz Biotechnology Inc. | WB (1:200) |

* IHC: immunohistochemistry; WB: western blot analysis.

† This antibody against Pak1 also cross-reacted with Pak2.
