# Supplementary material for: p21-Activated Kinases 1, 2 and 4 in Endometrial Cancers: Effects on Clinical Outcomes and Cell Proliferation
Source: PLoS One. 2015 Jul 28;10(7):e0133467. doi: 10.1371/journal.pone.0133467 (PMC4517872; doi:10.1371/journal.pone.0133467)
Supplement: S2 Table — (DOC) [file pone.0133467.s002.doc]

**S2 Table. Primers used for real-time PCR.**

| **Gene** | **Primer direction** | **Primer Sequence (5’ to 3’)** |
| --- | --- | --- |
| PAK1 | Forward | AGTTTCAGAAGATGAGGATGATGA |
|  | Reverse | AATCACAGACCGTGTGTATACAG |
| PAK4 | Forward | ATGTGGTGGAGATGTACAACAGCTA |
|  | Reverse | GTTCATCCTGGTGTGGGTGAC |
| CDC25A | Forward | AGATAGCAGTGAACCAGG |
|  | Reverse | TGCATCGGTTGTCAAGG |
| CYCLIN D1 | Forward | GCTGCTCCTGGTGAACAAGC |
|  | Reverse | TTCAATGAAATCGTGCGGG |
| ERα | Forward | ACCATGACCCTCCACACCAAAGCATC |
|  | Reverse | CAGCAAGCCCGCCGTGTAC |
| PR | Forward | GAGAGCTCATCAAGGCAATTGG |
|  | Reverse | TTCAATGAAATCGTGCGGG |
| GAPDH | Forward | TCCATGACAACTTTGGTATCGTG |
|  | Reverse | ACAGTCTTCTGGGTGGCAGTG |
