# Supplementary material for: p21-Activated Kinases 1, 2 and 4 in Endometrial Cancers: Effects on Clinical Outcomes and Cell Proliferation
Source: PLoS One. 2015 Jul 28;10(7):e0133467. doi: 10.1371/journal.pone.0133467 (PMC4517872; doi:10.1371/journal.pone.0133467)
Supplement: S3 Table — (DOC) [file pone.0133467.s003.doc]

| **Parameters** | **Case (n)** |  | **Mean ± SD** | **p-value** |  | **Mean ± SD** | **p-value** |  | **Mean ± SD** | **p-value** | **Mean ± SD** | **p-value** | **Mean ± SD** | **p-value** | **Mean ± SD** | **p-value** | **Mean ± SD** | **p-value** |
| --- | --- | --- | --- | --- | --- | --- | --- | --- | --- | --- | --- | --- | --- | --- | --- | --- | --- | --- |
| **Stage (FIGO)** |  |  |  |  |  |  |  |  |  |  |  |  |  |  |  |  |  |  |
| I | 44 |  | 10.56 ± 5.98 |  |  | 3.93 ± 3.47 |  |  | 2.55 ± 2.70 |  | 12.3 ± 3.57 |  | 7.84 ± 4.14 |  | 4.57 ± 4 |  | 11.66 ± 4.80 |  |
| II | 5 |  | 14.4 ± 2.19 |  |  | 3 ± 2 |  |  | 2.2 ± 1.30 |  | 11.2 ± 4.38 |  | 6 ± 5.66 |  | 4 ± 2.83 |  | 8.8 ± 4.66 |  |
| III | 10 |  | 11.44 ± 4.45 |  |  | 4.10 ± 3.70 |  |  | 1.7 ± 1.25 |  | 9.7 ± 3.50 |  | 6.3 ± 3.62 |  | 3.3 ± 3.62 |  | 9.3 ± 5.29 |  |
| IV | 2 |  | 5 ± 1.41 | 0.308* |  | 1 ± 1.41 | 0.498* |  | 1 ± 1.41 | 0.577* | 6 ± 2.83 | 0.053* | 3 ± 1.41 | 0.139* | 0 | 0.182* | 9 ± 4.24 | 0.286* |
| Early (I-II) | 49 |  | 10.96 ± 5.81 |  |  | 3.84 ± 3.35 |  |  | 2.51 ± 2.58 |  | 12.18 ± 3.62 |  | 7.65 ± 4.28 |  | 4.51 ± 3.88 |  | 11.37 ± 4.82 |  |
| Late (III-IV) | 12 |  | 10.42 ± 4.58 | 0.527† |  | 3.58 ± 3.58 | 0.68† |  | 1.58 ±1.24 | 0.209† | 9.08 ± 3.58 | 0.017† | 5.76 ± 3.55 | 0.145† | 2.75 ± 3.52 | 0.098† | 9.25 ± 4.96 | 0.162† |
|  |  |  |  |  |  |  |  |  |  |  |  |  |  |  |  |  |  |  |
| **Histological grade** |  |  |  |  |  |  |  |  |  |  |  |  |  |  |  |  |  |  |
| 1 | 25 |  | 12.08 ± 5.19 |  |  | 4.76 ± 3.876 |  |  | 3.12 ± 3.321 |  | 13.6 ± 3.096 |  | 8.84 ± 3.912 |  | 5.92 ± 4.3 |  | 12.92 ± 4.122 |  |
| 2 | 18 |  | 12.89 ± 3.95 |  |  | 4 ± 3.581 |  |  | 2.06 ± 1.259 |  | 11.39 ± 3.381 |  | 6.78 ± 4.333 |  | 2.06 ± 1.984 |  | 9.39 ± 5.066 |  |
| 3 | 18 |  | 7.06 ± 5.87 | 0.002* |  | 3.05±2.99 | 0.238* |  | 1.63±1.3 | 0.217* | 9.11 ± 3.494 | <0.001* | 6.21 ± 4.467 | 0.041* | 3.83 ± 3.484 | 0.003* | 10.11 ± 5.032 | 0.036* |
| Low (1-2) | 43 |  | 12.42 ± 4.68 |  |  | 4.44 ± 3.73 |  |  | 2.67 ± 2.69 |  | 12.67 ± 3.37 |  | 7.98 ± 4.17 |  | 4.30 ± 3.99 |  | 11.44 ± 4.82 |  |
| High (3) | 18 |  | 7.06 ± 5.87 | <0.001† |  | 3.05±2.99 | 0.129† |  | 1.63±1.3 | 0.119† | 9.11 ± 3.494 | 0.001† | 6.21 ± 4.467 | 0.072† | 3.83 ± 3.484 | 0.705† | 10.11 ± 5.032 | 0.358† |
|  |  |  |  |  |  |  |  |  |  |  |  |  |  |  |  |  |  |  |
| **Histological type** |  |  |  | . |  |  |  |  |  |  |  |  |  |  |  |  |  |  |
| Endometrioid | 53 |  | 12.38 ± 4.76 |  |  | 3.68 ± 3.429 |  |  | 2.38 ± 2.529 |  | 11.72 ± 3.914 |  | 7.4 ± 4.249 |  | 4.08 ± 3.852 |  | 11.17 ± 4.894 |  |
| Non-endometrioid | 11 |  | 3.33 ± 2.312 | <0.001† |  | 5.36 ± 3.722 | 0.118† |  | 2.27 ± 1.272 | 0.547† | 11.36 ± 2.908 | 0.666† | 7.55 ± 4.413 | 0.866† | 4.18 ± 5.656 | 0.878† | 10.91 ± 4.826 | 0.882† |
|  |  |  |  |  |  |  |  |  |  |  |  |  |  |  |  |  |  |  |
| **Myometrial invasion** |  |  |  |  |  |  |  |  |  |  |  |  |  |  |  |  |  |  |
| < 1/2 | 39 |  | 11.16 ± 5.731 |  |  | 4.79 ± 3.826 |  |  | 2.74 ± 2.531 |  | 12.33 ± 3.474 |  | 8 ± 4.525 |  | 4.56 ± 3.768 |  | 11.49 ± 4.695 |  |
| ≥ 1/2 | 17 |  | 11.25 ± 5.6 | 0.952† |  | 2.06 ± 2.015 | 0.005† |  | 1.24 ± 1.091 | 0.002† | 9.94 ± 4.264 | 0.041† | 6.18 ± 3.988 | 0.132† | 2.71 ± 2.910 | 0.083† | 9.76 ± 5.298 | 0.241† |
|  |  |  |  |  |  |  |  |  |  |  |  |  |  |  |  |  |  |  |
| **Involving cervix** |  |  |  |  |  |  |  |  |  |  |  |  |  |  |  |  |  |  |
| Negative | 49 |  | 10.92 ± 5.6 |  |  | 4.29 ± 3.488 |  |  | 2.59 ± 2.565 |  | 12.24 ± 3.443 |  | 7.78 ± 4.048 |  | 4.43 ± 3 .873 |  | 11.61 ± 4.604 |  |
| Positive | 15 |  | 10.93 ± 5.69 | 0.915† |  | 2.93 ± 3.494 | 0.064† |  | 1.6 ± 1.242 | 0.131† | 9.73 ± 4.148 | 0.029† | 6.27 ± 4.788 | 0.099† | 3.00 ± 3.402 | 0.182† | 9.53 ± 5.423 | 0.18† |
|  |  |  |  |  |  |  |  |  |  |  |  |  |  |  |  |  |  |  |
| **Age** |  |  |  |  |  |  |  |  |  |  |  |  |  |  |  |  |  |  |
| ≤ 57 | 30 |  | 12.8 ± 4.26 |  |  | 4.33 ± 3.97 |  |  | 2.87 ± 3.01 |  | 11.53 ± 3.66 |  | 7.5 ± 4.13 |  | 3.87 ± 3.67 |  | 11.87 ± 4.75 |  |
| > 57 | 37 |  | 8.7 ± 5.93 | 0.004† |  | 3.59 ± 3.12 | 0.589† |  | 1.89 ± 1.49 | 0.175† | 11.11 ± 4.23 | 0.769† | 6.89 ± 4.39 | 0.446† | 4.16 ± 3.84 | 0.818† | 10.38 ± 4.92 | 0.202† |
|  |  |  |  |  |  |  |  |  |  |  |  |  |  |  |  |  |  |  |

**S3 Table.** Correlation of Pak1, nuclear and cytoplasmic p-Pak2, Pak4 and p-Pak4 with clinicopathological parameters in endometrial cancer.

*Kruskal–Wallis rank test; †Mann-Whitney test; Those with significant P-values were underlined.
